# The Road to Metagenomics: From Microbiology to DNA Sequencing Technologies and Bioinformatics

**DOI:** 10.3389/fgene.2015.00348

**Published:** 2015-12-17

**Authors:** Alejandra Escobar-Zepeda, Arturo Vera-Ponce de León, Alejandro Sanchez-Flores

**Affiliations:** ^1^Unidad de Secuenciación Masiva y Bioinformática, Instituto de Biotecnología, Universidad Nacional Autónoma de MéxicoCuernavaca, México; ^2^Programa de Ecología Genómica, Centro de Ciencias Genómicas, Universidad Nacional Autónoma de MéxicoCuernavaca, México

**Keywords:** metagenomics, bioinformatics, high-throughput sequencing, taxonomy, functional genomics, microbiology

## Abstract

The study of microorganisms that pervade each and every part of this planet has encountered many challenges through time such as the discovery of unknown organisms and the understanding of how they interact with their environment. The aim of this review is to take the reader along the timeline and major milestones that led us to modern metagenomics. This new and thriving area is likely to be an important contributor to solve different problems. The transition from classical microbiology to modern metagenomics studies has required the development of new branches of knowledge and specialization. Here, we will review how the availability of high-throughput sequencing technologies has transformed microbiology and bioinformatics and how to tackle the inherent computational challenges that arise from the DNA sequencing revolution. New computational methods are constantly developed to collect, process, and extract useful biological information from a variety of samples and complex datasets, but metagenomics needs the integration of several of these computational methods. Despite the level of specialization needed in bioinformatics, it is important that life-scientists have a good understanding of it for a correct experimental design, which allows them to reveal the information in a metagenome.

## Brief history of microbial communities study

From various definitions of microbial communities, the one proposed by Begon et al. (1986) defines it as the set of organisms (in this case, microorganisms) coexisting in the same space and time. The study of microbial communities has changed from the first report of microbes made by Leeuwenhoek and their oral organisms in 1676 (Schierbeek, 1959), to the characterization using the current molecular techniques. Pioneer scientists tried to isolate these “invisible” organisms, and like Robert Koch, they started by using nutrients in a solid phase like potato slices or gelatine to cultivate and isolate microorganisms in order to count and visualize them. Ultimately, these isolation techniques helped scientists to understand the microorganisms' physiologies (Blevins and Bronze, 2010).

Soon, the microscope became the principal tool to study microorganisms and their interactions. Development of practical staining techniques such as Gram, Ziehl–Neelsen, and Schaeffer and Fulton (Beveridge, 2001; Blevins and Bronze, 2010) significantly improved the resolution of microscopy techniques. Something evident to microbiologist was that the number of observed microorganisms in a microscope did not correspond with number of microorganism obtained in culture plates (Staley and Konopka, 1985). Although the explanation to this observation was not evident at that time, the conclusion was that the microorganisms need special conditions to grow, and based on this, Winogradsky emulated environments for culture media production that resembled native growing conditions (McFall-Ngai, 2008). Winogradsky's ideas and his contribution to ecology revolutionized microbiology and gave birth to a new concept named “microbial ecology,” which refers to the study of microorganisms and their environmental roles (Ackert, 2012).

**Figure 1:**
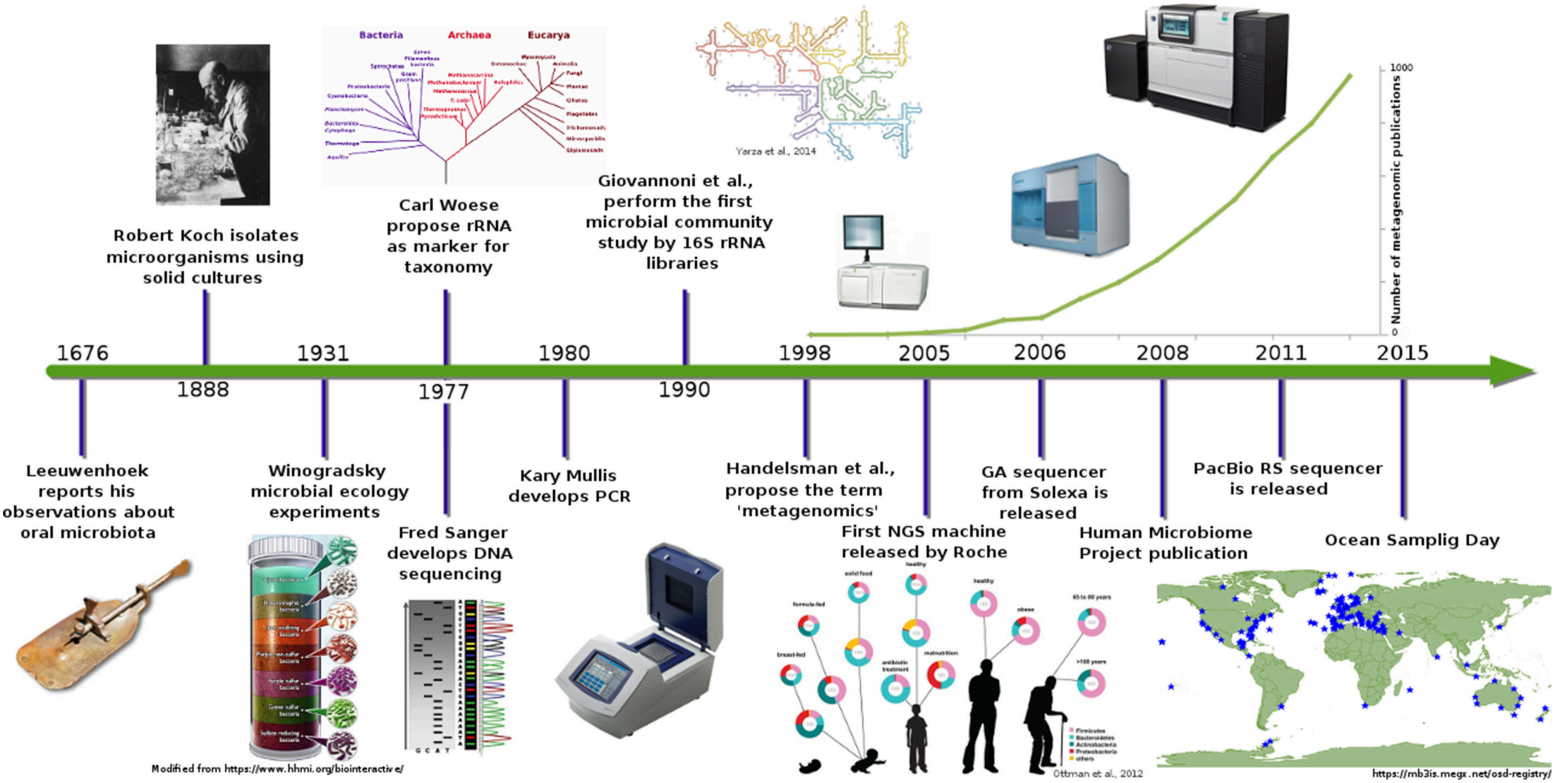
**Metagenomics timeline and milestones**. Timeline showing advances in microbial communities studies from Leeuwenhoek to NGS (Ottman et al., 2012; Yarza et al., 2014).

For almost 300 years (Figure 1), the study of microorganisms was based on morphology features, growth, and selection of some biochemical profiles (Roszak et al., 1984; Oliver et al., 1991; Colwell et al., 1996). These techniques provided an insight into the microbial world, but nowadays, they provide only a limited resolution for other applications.

In the late 1970s, Carl Woese proposed the use of ribosomal RNA genes as molecular markers for life classification (Woese and Fox, 1977). This idea in conjunction with the Sanger automated sequencing (Sanger et al., 1977) method revolutionized the study and classification of microorganisms. Some decades later, advances in molecular techniques were applied to microbial diversity description and granted access to a “new uncultured world” of microbial communities. Some of these techniques, which had a remarkable impact, were the polymerase chain reaction (PCR), rRNA genes cloning and sequencing, fluorescent *in situ* hybridization (FISH), denaturing gradient gel electrophoresis (DGGE and TGGE), restriction-fragment length polymorphism, and terminal restriction-fragment length polymorphism (T-RFLP). However, in spite all these improvements, there were many other observations in microbiology that remained unanswered like those related to the microorganisms' metabolic and ecological function. Characterization of certain functions in a particular environment was possible only after gene cloning from total DNA of a certain habitat and when its heterologous expressed product was associated with a given metabolic function (i.e., nitrogenases, cellulases, oxidoreductases, laccases, etc.). This implied the development of gene expression techniques using other microorganisms as systems to test gene function and roles in the microbial community. In addition, a window of opportunity was open to discover new genes, functions, and metabolic products with technological application, thereby giving birth to biotechnology. Products such as “terragines” from *Streptomyces lividians* (Wang et al., 2000) or genes related to broad-spectrum antibiotics were cloned from soil-DNA libraries (Gillespie et al., 2002) were achievements that set the foundation to a new area named “metagenomics analysis,” which was later defined as the theoretical collection of all genomes from members in a microbial community from a specific environment (Handelsman et al., 1998). Even if these approaches led to the discovery of new molecules and identification of new microbial communities members (Giovannoni et al., 1990), more recently, some problems have been spotted. Cloning biases (Morgan et al., 2010), sampling biases, misidentification of “decorating enzymes” and incorrect promoter sites in genomes, and dispersion of genes involved in secondary metabolite production (Keller and Zengler, 2004) are some of the problems found in metagenomics. Therefore, it is important to evaluate and correct these biases with statistical methods to have a better understanding of the species richness and know the difference between the expected and the observed microbial diversity.

## Concepts of microbial diversity and species richness

“Species diversity” is an attribute of any biological community (Krebs, 2014), but how we quantify it, is not trivial. The simplest idea to describe and quantify a microbial community (e.g., a metagenome) is the species richness concept, which refers to the number of species in a specified region. Another idea that can be applied to metagenomics is the evenness concept or differential abundance proposed by Simpson (1949). The evenness measurement attempts to quantify the unequal representation in communities where there are few dominant species and many species that are relatively uncommon. This could be tested against a hypothetical community in which all species are equally common. Therefore, when comparing two communities, if both have the same number of species (equal species richness) but different abundances, then the consortia with the shortest difference between the observed and hypothetical distribution (even abundance) will be the more diverse. Hence, it should be considered that species richness should not be the only parameter to define diversity.

In order to describe and compare communities in a better way, there are other metrics that have been adapted to metagenomics and that can complement the aforementioned. Alpha (α) is a metric for local diversity of a community; opposite to it, we have Gamma (γ), which measures the total regional diversity that includes many communities, and finally Beta (β) metric tells us how different community samples are in an area, linking Alpha and Gamma metrics (Krebs, 2014).

In the Alpha diversity assessment, the accumulation of species or Operational Taxonomic Units (OTUs) plots have been used to evaluate the sample efficiency and to correct sampling problems. Although a species accumulation curve could present an asymptotic trend after using a bigger sample size, the maximum species number could not be reached. This is why a statistical approach has to be performed, i.e., rarefaction curves, which are useful to estimate the real maximum species or OTUs number observed in the sample and to compare samples with different sizes (Sanders, 1968; Heck et al., 1975; Colwell and Coddington, 1994).

Another alternative to calculate species diversity quantitatively is the use of statistical estimators. Particularly, non-parametric estimators have been used for microbial communities' studies. These estimators do not depend on the statistical behavior of the sample and can consider low abundance species. On one hand, the simplest non-parametric diversity estimator is the Simpson's index (*D*), which is based on the probability of assigning two independent individuals taken randomly from the community into the same species (Simpson, 1949). On the other hand, Shannon–Wiener function or Shannon–Weaver index *H*′ (Shannon, 1948) is an entropy measurement that increases with the number of species in the sample. Simpson and Shannon–Wiener indices are used as heterogeneity measurements and differ mainly in calculation of the taxa abundance for the final richness estimation. Simpson index gives a higher weight to species with more frequency in a sample, whereas Shannon–Wiener gives more weight to rare species (Krebs, 2014).

The development of molecular biology provided a new vision of microbial ecology and allowed the study of highly complex communities in a short period of time. However, the application of diversity estimators in metagenomics projects has been evaluated by some authors with divided ideas about their results.

Some authors concluded that microbial diversity estimation based on molecular markers is possible and can be used for comparison with some precautions (Gihring et al., 2012). They recommended the use of Simpson or Shannon–Wiener estimators as the best descriptors for species richness at high-level taxa in metagenomes (Haegeman et al., 2013; Chernov et al., 2015). However, in nature, the microbial communities have a large number of rare species that can be detected only if an exhaustive sampling is performed (Colwell and Coddington, 1994; Kemp and Aller, 2004; Bonilla-Rosso et al., 2012). Therefore, the use of such estimators is unsuccessful for very complex microbial communities. This problem has generated the creation of new diversity indexes for species that analyse statistically the behavior of the sample. For example, the tail statistic (τ) estimates the number of undiscovered species from a rank abundance curve, giving a higher weight to the low abundant taxa and increasing the sensitivity of the analysis of complex samples (Li et al., 2012).

The use of diversity indexes is a better approach to quantify and compare microbial diversity among samples. Such comparison should be done cautiously because it could be uninformative unless biases related to sampling and criteria for species or OTU definition are minimized (Bonilla-Rosso et al., 2012).

## Next generation sequencing technologies to explore microbial communities

As previously mentioned, Sanger sequencing technology had a great impact on the early stage of microbial community studies. Nowadays, the sequencing yield and sequence length have changed a lot since Sanger sequencing (Table 1). Currently, Sanger sequencing can retrieve up to 96 sequences per run with an average length of 650 bp, which might be enough for phylogenetic marker analysis. However, low-cost platforms known as Next Generation Sequencing technologies (NGS) are capable of parallel sequencing millions of DNA molecules with different yields and sequence lengths (Table 1; Logares et al., 2012; Fichot and Norman, 2013; Glenn, 2014; Sanchez-Flores and Abreu-Goodger, 2014) having a positive impact in different areas.

**Table 1:** **Direct comparison among sequencing technologies suitable for metagenomics**.

	**Roche 454**	**IonTorrent PGM**	**Illumina**	**PacBio RSII^a^**
Maximum read length (bp)	1200	400	300^b^	50,000
Output per run (Gb)	1	2	1000^c^	1
Amplification for library construction	Yes	Yes	Yes	No
Cost/Gb (USA Dollar)	$9538.46	$460.00	$29.30	$600
Error kind	Indel	Indel	Substitution	Indel
Error rate (%)	1	~1	~0.1	~13
Run time	20 h	7.3 h	6 days	2 h

Adapted from Glenn, T. 2014 NGS Field Guide—Table 2a—Run time, Reads, Yield|The Molecular Ecologist. Available online at:

*http://www.molecularecologist.com/next-gen-fieldguide-2014/ (Accessed Aug 17, 2015)*.

a *P6-C4 chemistry*.

b *MiSeq read length*.

c *Illumina HiSeq 2500 Dual flowcell yield*.

The first of these technologies that revolutionized the genomics and metagenomics areas was the 454 sequencing platform or “pyrosequencing.” The principle of this technology is a one-by-one nucleotide addition cycle, where the pyrophosphate (PPi) released from the DNA polymerization reaction is transformed in a luminous signal. The light emission from a plate with millions of microwells containing a given DNA fragment is detected by the machine and is translated to nucleotide sequences with an associated base quality value (Margulies et al., 2005). This technology offered a higher yield than Sanger sequencing at a lower cost but with shorter read lengths (Table 1). The main bias of this technology is artificial insertions and deletions due to long homopolymeric regions. In spite of the advantages that this technology provided to metagenomics, it is now obsolete. Recent announcements by Roche (current owner of the technology) reported the shutdown of 454 division, ceasing the platform support by mid-2016 (Karow, 2013). Nevertheless, all the software that has been developed so far to analyse 454 data could be adapted to analyse data obtained by another platforms.

The Ion Torrent platform is an analogous technology to 454 that produces a similar yield and a read length to those obtained at its middle stage of development. The Ion Torrent PGM is considered as the smallest potentiometer that exists and can detect the change in hydrogen potential generated each time a proton is released after a nucleotide is added in the sequencing reaction occurring in millions of microwells (Rothberg et al., 2011). The maximum Ion Torrent yield is ~500 million reads with a mode length of 400 bp (Table 1) (Glenn, 2014). In this case, there is a clear benefit in terms of cost reduction, since Ion Torrent sequencing is just a tenth of the pyrosequencing cost (Whiteley et al., 2012).

However, read length reduction in return for higher yields and error-rates is another trade-off observed in some platforms in order to reduce the sequencing costs, i.e., the case of the Illumina technology, which has become one of the most popular technologies due to its low cost and high yield. The basis of Illumina chemistry is the reversible-termination sequencing by synthesis with fluorescently labeled nucleotides. In a nutshell, DNA fragments are attached and distributed in a flow cell, where the sequencing reaction occurs by adding a labeled nucleotide. When the labeled nucleotide is incorporated and its fluorescent molecule is excited by a laser, the signal is registered by the machine. Afterwards, the fluorophore molecule is removed and the next nucleotide can be incorporated. DNA fragments can be sequenced from one or both sides giving single end or pair-end sequencing, respectively, with a maximum read length of 300 base pairs per read (Bennett, 2004). The output of this technology is currently the highest among the second generation sequencing technologies and makes it suitable for multiplexing hundreds of samples (Table 1; Glenn, 2014).

Currently, the technologies already mentioned are the most used for metagenome projects, but the development of sequencing was kept going for the last 5 years in order to solve the known biases of these technologies and to offer a better trade-off between yield, cost, and read length. At present, the so called third generation sequencing technologies such as PacBio RS from Pacific Bioscience (Fichot and Norman, 2013) or the Oxford Nanopore (Kasianowicz et al., 1996), which are single-molecule, real-time technologies, reduced the amplification bias and also the short read length problem. The time and cost reduction offered by these technologies is also a valuable asset. However, the error rate is higher compared to other technologies but correctable if the sequencing depth is high enough. In terms of computational tools, there is virtually no software that can be used for metagenomics analysis.

One of the great improvements of second and third generation sequencing technologies is that the library preparation does not require DNA cloning vectors or bacterial hosts, simplifying the library preparation and reducing DNA contamination from other organisms that are not part of the metagenome.

Although new generation sequencing technologies are powerful and have allowed us to discover novel microbial worlds and explore new environments, they present particular limitations and biases that have to be circumvented (Table 1). It is important to consider that data obtained from second or third generation sequencing technologies have certain computational requirements for their analysis. The bigger the dataset generated, the higher computational resources and more complex bioinformatics analyses are necessary. In addition, large data storage is needed to archive and process the data (Logares et al., 2012). In terms of bioinformatic analysis, not only high-end servers are required but also UNIX operative system skills are needed. Programming and scripting knowledge are desirable to run and install the available metagenomics software for parsing and interpreting the results. Thus, it is suggested that biologists or biological scientists should develop basic computational skills in order to take an advantage of metagenomic data.

### Quality control (QC) procedures for metagenomics

Assessing the output quality from any of the previously mentioned sequencing technologies will be always a crucial step before starting any analysis. Each sequencing platform presents a particular bias product of the intrinsic mechanism to detect each nucleotide, which conforms the DNA polymer that is being analyzed (Table 1). The error rate from each technology varies, affecting the characterization of a microbial community (Luo et al., 2012). Filtering low quality reads considerably improves metagenome analyses such as taxonomical classification and α and β diversity calculation (Bokulich et al., 2013). There are several programs that can be used for sequencing read QC analysis as described in Table 1. In general, they provide information about the sequencing output (number or reads, length, GC content, overrepresented sequences, etc.) and some of them include tools to modify the reads (adapter removal, quality filtering or trimming). These QC operations need an interpretation depending on the analysis. For example, a GC content analysis can be used to anticipate the presence of organisms with different GC content, but a single GC distribution does not imply that our sample has very low diversity, just a bias toward the GC content of the most abundant organisms. Removal of low quality bases or entire reads can be beneficial in terms of mapping, but for metagenome assembly (or any other genome assembly), none of the current assembly programs use or interpret base quality within the assembly process. For Illumina sequencing, removal of optical or PCR duplicates can increase the quality of abundance analysis from whole metagenome shotgun DNA sequencing. However, this QC control has no sense at all in amplicon sequence analysis. Therefore, there are some compulsory QC processes that need to be performed before analysing our data, but depending on the approach, we have to design specific QC steps to improve our results.

## Reconstructing the genomic content of the microbial community from NGS data

The main questions to answer in microbial ecology are “Who is out there?” and “What are they doing?” In fact, metagenomics can answer both questions. Particularly, microbial diversity can be determined using two different approaches: (1) Amplicon sequencing or (2) Shotgun metagenomics. In the first approach, specific regions of DNA from communities are amplified using taxonomical informative primer targets such as 16S rRNA gene for prokaryotes and intergenic transcribed spacers (ITS) or the large ribosomal subunit (LSU) gene for eukaryotes (Sharpton, 2014; Tonge et al., 2014). In the second approach, shotgun metagenomics can help to reconstruct large fragments or even complete genomes from organisms in a community without previous isolation, allowing the characterization of a large number of coding and non-coding sequences that can be used as phylogenetic markers.

### Amplicon sequencing analysis

First of all, the term “metagenomics” should not be used to refer amplicon sequence analysis, as this analysis is based on just one gene instead of the collection of all the genes in the available genomes from all the organisms in a sample. A better term proposed is “metaprofiling,” and it should be interpreted in the rest of this text as the study of all members in a microbial community based on one gene or marker (i.e., 16S rRNA gene) for taxonomy or phylogenetic purposes.

Metaprofiling has been widely used due to its convenience to perform taxonomic and phylogenetic classification in large and complex samples within organisms from different life domains. In addition, it could be performed using almost all mentioned sequencing technologies (Table 1).

Moneywise, metaprofiling is currently the best option for 16S rRNA amplicon library preparation and sequencing by platforms such as the Illumina MiSeq or the Ion Torrent PGM. These benchtop sequencers allow microbial ecologists to perform diversity studies at their labs, using multiple replicates and samples from longitudinal time studies. Previous comparisons between HiSeq 2000 and MiSeq technologies have shown that despite the yield difference between them (>50 Gb per day against 5 Gb), the number of OTUs obtained are not significantly different on using both the technologies (Caporaso et al., 2012; Luo et al., 2012).

The advantages of amplicon sequencing are contrasted by the bias generated from using only one phylogenetic marker such as the 16S ribosomal gene or a variable region from it. Some of the pitfalls are low resolution at the species level (Petrosino et al., 2009; Nalbantoglu et al., 2014), a range in gene copy number in many species (Acinas et al., 2004), horizontal transfer of 16S rRNA genes (Schouls et al., 2003; Bodilis et al., 2012), and the fact that < 0.1% of the total genome are ribosomal genes, hindering the amplification of this marker from very low abundant genomes in a sample.

The ribosomal genes as phylogenetic markers have been used for the last 40 years or so, resulting in a wide representation of this marker in many databases, allowing the taxonomic annotation of almost any microorganisms present in a metagenomic sample. Some database examples are Greengenes (DeSantis et al., 2006), the Ribosomal Database Project (Wang et al., 2007), and Silva (Quast et al., 2013). The latter includes a great catalog of eukaryotic LSU sequences and is convenient to analyse fungi or other metazoan microorganisms. However, amplicon-dependent techniques are prone to sequencing errors, such as result discrepancy from using different ribosomal variable regions, primers bias, and OTU assignment errors (Fox et al., 1992; Logares et al., 2012; Poretsky et al., 2014).

Most of the earlier amplicon analysis programs were designed for Sanger or 454 ribosomal pyrotag sequences. For example, Mothur (Schloss et al., 2009), QIIME (Caporaso et al., 2010), MEGAN (Huson and Weber, 2013), and CARMA (Krause et al., 2008) are some of the legacy software still available. Nowadays, the software development for metagenomics considers short sequences like Illumina reads or very long sequences such as PacBio reads (Table 2).

**Table 2:** **Examples of software used in metagenomic and metaprofiling analysis**.

**Software**	**Application**	**References**	**Link (website)**
FastQC	Quality control tool for high-throughput sequence data using modular options and giving graphic results of quality per base sequence, GC content, N numbers, duplication, and over represent	Andrews, 2015	http://www.bioinformatics.babraham.ac.uk/projects/fastqc/
Fastx-Toolkit	Command line tools for Short-reads quality control. These allow processing, cutting, format conversion, and collapsing by sequence length and identity	NP	http://hannonlab.cshl.edu/fastx_toolkit/index.html
PRINTSEQ	Quality control tool for sequence trimming based in dinucleotide occurrence and sequence duplication (mainly 5′/3′)	Schmieder and Edwards, 2011	http://prinseq.sourceforge.net/
NGS QC Toolkit	Tool for quality control analysis performed in parallel environment	Patel and Jain, 2012	http://www.nipgr.res.in/ngsqctoolkit.html
Meta-QC-Chain	Parallel environment tool for quality control. This performs a mapping against 18S rRNA databases for removing eukaryotic contaminant sequences	Zhou et al., 2014	http://www.computationalbioenergy.org/qc-chain.html
Mothur	From reads quality analysis to taxonomic classification, calculus of diversity estimators and ribosomal gene metaprofiling comparison	Schloss et al., 2009	http://www.mothur.org/
QIIME	Quality pre-treatment of raw reads, taxonomic annotation, calculus of diversity estimators, and comparison of metaprofiling or metagenomic data	Caporaso et al., 2010	http://qiime.org/
MEGAN	Taxonomy and functional analysis of metagenomic reads. It based on BLAST output of short reads and performs comparative metagenomics. Graphical interface	Huson and Weber, 2013	http://ab.inf.uni-tuebingen.de/software/megan5/
CARMA	Phylogenetic classification of reads based on Pfam conserved domains	Krause et al., 2008	http://omictools.com/carma-s1021.html
PICRUSt	Predictor of metabolic potential from taxonomic information obtained of 16S rRNA metaprofiling projects	Langille et al., 2013	http://picrust.github.io/picrust/
Parallel-meta	Taxonomic annotation of ribosomal gene markers sequences obtained by metaprofiling or metagenomic reads. Functional annotation based on BLAST best hits results. Comparative metagenomics	Su et al., 2014	http://www.computationalbioenergy.org/parallel-meta.html
MOCAT	Pipeline that includes quality treatment of metagenomic reads, taxonomic annotation based on single copy marker genes classification, and gene-coding prediction	Kultima et al., 2012	http://vm-lux.embl.de/~kultima/MOCAT2/index.html
TETRA	Taxonomic classification by comparison of tetranucleotide patterns. Web service available	Teeling et al., 2004	http://omictools.com/tetra-s1030.html
PhylophytiaS	Composition-based classifier of sequences based on reference genomes signatures	McHardy et al., 2007	http://omictools.com/phylopythia-s1455.html
MetaclusterTA	Taxonomic annotation based on binning of reads and contigs. Dependent of reference genomes	Wang et al., 2014	http://i.cs.hku.hk/~alse/MetaCluster/
MaxBin	Unsupervised binning of metagenomic short reads and contigs	Wu et al., 2014	http://sourceforge.net/projects/maxbin/
Amphora and Amphora2	Metagenomic phylotyping by single copy phylogenetic marker genes classification	Wu and Eisen, 2008; Wu and Scott, 2012	http://pitgroup.org/amphoranet/
BWA	Algorithm for mapping short-low-divergent sequences to large references. Based on Burrows–Wheeler transform	Li and Durbin, 2009	http://bio-bwa.sourceforge.net/
Bowtie	Fast short read aligner to long reference sequences based on Burrows–Wheeler transform	Langmead and Salzberg, 2012	http://bowtie-bio.sourceforge.net/index.shtml
Genometa	Taxonomic and functional annotation of short-reads metagenomic data. Graphical interface	Davenport and Tümmler, 2013	http://genomics1.mh-hannover.de/genometa/
SORT-Items	Taxonomic annotation by alignment-based orthology of metagenomic reads	Monzoorul Haque et al., 2009	http://metagenomics.atc.tcs.com/binning/SOrt-ITEMS
DiScRIBinATE	Taxonomic assignment by BLASTx best hits classification of reads	Ghosh et al., 2010	http://metagenomics.atc.tcs.com/binning/DiScRIBinATE/
IDBA-UD	Assembler *de novo* of metagenomic sequences with uneven depth	Peng et al., 2012	http://i.cs.hku.hk/~alse/hkubrg/projects/idba_ud/
MetaVelvet	*De novo* assembler of metagenomic short reads	Namiki et al., 2012	http://metavelvet.dna.bio.keio.ac.jp/
Ray Meta	Assembler of *de novo* of metagenomic reads and taxonomy profiler by Ray Communities	Boisvert et al., 2012	http://denovoassembler.sourceforge.net/
MetaGeneMark	Gene coding sequences predictor from metagenomic sequences by heuristic model	Zhu et al., 2010	http://exon.gatech.edu/index.html
GlimmerMG	Gene coding sequences predictor from metagenomic sequences by unsupervised clustering	Kelley et al., 2012	http://www.cbcb.umd.edu/software/glimmer-mg/
FragGeneScan	Gene coding sequences predictor from short reads	Rho et al., 2010	http://sourceforge.net/projects/fraggenescan/
CD-HIT	Clustering and comparing sequences of nucleotides or protein	Li and Godzik, 2006	http://weizhongli-lab.org/cd-hit/
HMMER3	Hidden Markov models applied in sequences alignments	Eddy, 2011	http://hmmer.janelia.org/
BLASTX	Basic local alignment of translated sequences	Altschul et al., 1997	http://blast.ncbi.nlm.nih.gov/blast/Blast.cgi?PROGRAM=blastx&PAGE_TYPE=BlastSearch&LINK_LOC=blasthome
MetaORFA	Assembly of peptides obtained from predicted ORFs	Ye and Tang, 2008	NA
MinPath	Reconstruction of pathways from protein family predictions	Ye and Doak, 2009	http://omics.informatics.indiana.edu/MinPath/
MetaPath	Identification of metabolic pathways differentially abundant among metagenomic samples	Liu and Pop, 2011	http://metapath.cbcb.umd.edu/
GhostKOALA	KEGG's internal annotator of metagenomes by k-number assignment by GHOSTX searches against a non-redundant database of KEGG genes	NP	http://www.kegg.jp/ghostkoala/
RAMMCAP	Metagenomic functional annotation and data clustering	Li, 2009	http://weizhong-lab.ucsd.edu/rammcap/cgi-bin/rammcap.cgi
ProViDE	Analysis of viral diversity in metagenomic samples	Ghosh et al., 2011	http://metagenomics.atc.tcs.com/binning/ProViDE/
Phyloseq	Tool-kit to row reads pre-processing, diversity analysis and graphics production. R, Bioconductor package	McMurdie and Holmes, 2014	https://joey711.github.io/phyloseq/
MetagenomeSeq	Analysis of differentially abundance of 16S rRNA gene in metaprofiling data. R, Bioconductor package	Paulson et al., 2013	http://bioconductor.org/packages/release/bioc/html/metagenomeSeq.html
ShotgunFunctionalizeR	Metagenomic functional comparison at level of individual genes (COG and EC numbers) and complete pathways. R, Bioconductor package	Kristiansson et al., 2009	http://shotgun.math.chalmers.se/
Galaxy portal	Web repository of computational tools that can be run without informatic expertise. Graphical interface and free service	Goecks et al., 2010	https://usegalaxy.org/
MG-RAST	Taxonomic and functional annotation, comparative metagenomics. Graphical interface, web portal, and free service	Meyer et al., 2008	http://metagenomics.anl.gov/
IMG/M	Functional annotation, phylogenetic distribution of genes and comparative metagenomics. Graphical interface, web portal, and free service	Markowitz et al., 2012	https://img.jgi.doe.gov/cgi-bin/m/main.cgi

*NP, Not published in an indexed Journal; NA, Not web site available*.

Once the species level taxonomic annotation objective is covered, metagenome projects can focus on the functional information mining. This could be achieved from the taxonomical information by extrapolating the functional annotation of related reference genomes (De Filippo et al., 2012). To our knowledge, PICRUSt (Langille et al., 2013) is the only available software that connects the taxonomic classification from metaprofiling results with metabolic information (Table 2). PICRUSt uses an evolutionary modeling to generate functional predictions from ribosomal (16S rRNA) genes databases, which allows to obtain a general vision of microbial functions in a microbiome. However, it only works adequately for those environments where the results have large numbers of organisms with annotated reference genomes available. Finally, PICRUSt is only designed to analyse prokaryotes, ignoring a large amount of metabolic features performed by eukaryotes.

### Shotgun metagenomics

As mentioned, after deciphering the microbial diversity of a metagenome, it would be very convenient to understand its metabolic potential. This can be achieved by using a whole metagenome approach where total DNA is obtained to prepare whole shotgun libraries. As discussed, the sequencing platform choice will be somehow influenced by the computational resources and available software to handle and process the sequencing output (Table 2). It should be noted that the impact and potential of shotgun metagenomics would be also reflected in taxonomy species level classification. The many microorganisms obtained from whole metagenome shotgun sequencing will probably deliver new genes with novel functions.

#### Assessment of taxonomy based on markers

Theoretically, when a whole metagenome shotgun sequencing approach is performed, we can obtain a representation of all the genomes in the sample. This permit us not only to choose from a wide range of phylogenetic markers in order to perform taxonomic annotation but also we can obtain the ribosomal markers or any other used in the amplicon sequencing approach.

A multithreading software option to extract ribosomal marker genes from metagenomic sequences to conduct the taxonomic annotation is Parallel-meta (Su et al., 2014). The program collects ribosomal sequences from short reads by using a Hidden Markov Models (HMM)-based reconstruction algorithm (De Fonzo et al., 2007). Then it maps the reconstructed sequences to different 16S gene databases using Megablast (http://www.ncbi.nlm.nih.gov/blast/html/megablast.html). As discussed in the metaprofiling analysis section, taxonomical annotation could be improved by using more than one phylogenetic marker. Therefore, in whole metagenome shotgun sequencing, we can use software to search single copy marker genes in other databases. Two examples of programs using these approaches are MOCAT (Kultima et al., 2012), which uses the RefMG database (Ciccarelli et al., 2006) constituted by a collection of 40 single copy marker genes, and AMPHORA (Wu and Eisen, 2008), which includes a database containing around 31 single copy universal markers (Table 2). After the single copy marker identification, such pipelines perform an OTU multiple sequence alignment, distance calculation, and clustering. Finally, the taxonomical annotation is performed using reference genomes giving a species resolution in many cases.

#### The binning strategy

Binning classification is a quick and handy method to predict taxonomical composition using the information contained in the reads. These could be performed using either reads or assembled sequences. Binning algorithms use different strategies to get the taxonomic assignment: (a) sequence composition classification or (b) sequence alignment against references.

The first one is based on k-mer frequencies methods, which uses short words (k-mers) to represent a vector-like sequence and then to obtain the similarity among all words in the query. This representation can be considered as a “genomic signature” and was widely used by Karlin and Burge (1995) to explore evolutionary conservation among species. Examples of software that perform sequence classification by composition are TETRA (Teeling et al., 2004), PhylophytiaS (McHardy et al., 2007), and MetaclusterTA (Wang et al., 2014) (Table 2).

Other methods have more than one strategy to support the correct binning of sequences as in the case of MaxBin (Wu et al., 2014) and Amphora2 (Wu and Scott, 2012), which rely on finding single copy marker genes, k-mer signatures, GC content, and coverage information to perform contig and read binning.

In spite of the binning approach facilitating taxonomic classification, it should be considered that this strategy have some problems with horizontally transferred sequences, where genes from an organism appear in another. This could lead to an aggravated misclassification if it occurs between non-described organisms (Sharpton, 2014).

However, other methods based on reference read alignment are based on Burrows–Wheeler Transform indexes like BWA (Li and Durbin, 2009) or Bowtie (Langmead and Salzberg, 2012). These fast and accurate alignment methods can assess species richness and abundance in metagenomes by mapping reads directly to individual reference genomes or many concatenated genomes (pangenomes) or sequences. This last approach is used in the Genometa software (Davenport and Tümmler, 2013) and allows us to obtain OTUs for metagenome samples by grouping genomic islands, operons, or orthologous genes present in reference pangenomes. Furthermore, if long reads are available, then it is possible to do a taxonomic assignment by translating them and use all potential coding sequences to perform searches in annotated protein databases using local alignment tools, i.e., BLAST. In addition, some programs like SORT-Items (Monzoorul Haque et al., 2009), Megan, or Discribinate (Ghosh et al., 2010) (Table 2) can recover the lowest common ancestor (LCA) of a certain sequence from BLAST results.

Finally, we should consider that the more information we have for supporting taxonomic or functional results, the more reliable will be our conclusions. This is why it is always advisable to use more than one approach to assess taxonomic or functional annotation, if possible.

#### Functional metagenomics analysis

Reconstruction of metabolic pathways from enzyme-coding genes is a relevant matter in the metagenome analysis. Generally, there are two options to perform functional annotation from shotgun sequences, one is using sequencing reads directly and another is by read assembly.

##### Read assembly

Assembly is more efficient for genome reconstruction in low complex samples and when closely related species reference genomes are present (Teeling and Glöckner, 2012; Luo et al., 2013). However, the task is hampered when the read coverage is low and when there is high frequency of polymorphisms and repetitive regions (De Filippo et al., 2012). Nowadays, there are *ad hoc* assemblers for metagenome reads (Table 2) such as IDBA-UD (Peng et al., 2012) and MetaVelvet (Namiki et al., 2012). Both are based on de Bruijn graph construction methods and consider different coverage peaks, which are expected in a community composed by several different organisms (Thomas et al., 2012).

An extension of this algorithm is the use of the so called “colored” de Brujin graphs. This computational implementation can perform a genome assembly and variant calling at the same time (Iqbal et al., 2012). An assembler that incorporates this technique is Ray genome assembler that presents a different implementation such as RayMeta for *de novo* assembly of metagenomes and RayCommunities that calculates microbe abundance and taxonomical profiling (Table 2) (Boisvert et al., 2012).

Some advantages of assembling metagenomes are: (1) The possibility of analysing the genome context (i.e., operons); (2) Increasing the probability of complete genes and genomes reconstruction, arising the confidence of sequence annotation; (3) Analysis simplification by mapping long contigs instead of short reads (Thomas et al., 2012; Luo et al., 2013; Segata et al., 2013).

##### Prediction of gene coding sequences

After metagenome assembly, gene prediction and annotation are similar to the framework followed in whole genome characterization (Yandell and Ence, 2012; Richardson and Watson, 2013). For metagenomics, it is recommended to predict genes using algorithms that consider di-codons frequency, preferential bias in codon usage, patterns in the use of start and stop codons and, if possible, incorporates the information of species-specific ribosome-binding sites patterns, Open Reading Frame (ORF) length, and GC content of coding-sequences (Liu et al., 2013).

To assess such tasks, some gene predictors have been designed particularly for metagenomic contig ORFs calling (Table 2). For example, MetaGeneMark (Zhu et al., 2010) or GlimmerMG (Kelley et al., 2012) uses *ab initio* gene identification by “heuristical model” methods and second-order Markov chains for coding-sequence prediction training.

However, it is not always possible to get a good assembly, especially for complex metagenomes with a great number of low abundance species. A workaround would be the use of FragGeneScan tool, which predicts partial ORFs from short reads of at least 60 bp length (Rho et al., 2010).

With predicted genes, we can continue to analyse the translations of such predictions and obtain a product and functional annotation.

##### Function assignment and databases

Function assignment of predicted ORFs could be performed on either nucleotide or translated sequences. In both cases, homology detection is probably the easiest and most frequent annotation method, despite being computationally demanding and time consuming. Using algorithms like BLAST against databases such as Swiss-Prot or NCBI-nr retrieve a list of related hits with a certain annotation that can be used to mine taxonomical information as well. However, a limitation of this approach is the size and phylogenetic coverage of the database (Carr and Borenstein, 2014).

Searches in customized databases such as CAZY, dbCAN, or MetaBioMe are alternative to avoid time consuming and the use of excessive computational resources in the annotation of genes related to a metabolic pathway (Teeling and Glöckner, 2012; Yang et al., 2014). In any case, reducing computational workload is useful to remove redundant sequences using algorithms such as CD-HIT (Li and Godzik, 2006) to make the ORF or read annotation process more efficient.

Usually, when protein function assignment by homology is not possible due to low sequence identity values (< 20% of identity), HMM searches (Eddy, 2011) can be used for interrogating protein functional domain profiles using databases like the Conserved Domain Database of NCBI, PFAM, or SEED. Apart from solving the remote homology problem, this approach has helped us to find the regional or functional domains in proteins, in addition to the product annotation that sometimes could be cryptic.

Homology-based or HMM strategies can deliver a great number of false negatives especially when using short reads (Scholz et al., 2012; Yang et al., 2014). It is noteworthy that for functional annotation, the longer the sequence, the more information is provided, which makes the sequence search easier (Carr and Borenstein, 2014). The use of short reads to perform direct searches has low sensitivity and specificity for homologous identification (Wommack et al., 2008); therefore, *E*-value threshold should be adjusted in order to obtain correct results (Carr and Borenstein, 2014).

Another option is sequence clustering using BLASTX (Altschul et al., 1997). This strategy allows us to search directly from reads or contigs, since the program will perform all the possible translations. This has been implemented by Ye and Tang (2008) in the MetaORFA pipeline, where the translations (ORFome) are used to search homologs in the databases (Table 2). However, this could be very inefficient if a large set of reads is being analyzed.

A workflow summary for functional annotation could be as follows: get the best possible metagenome assembly (highest N50, N90, and contig/scaffold ave. length) to perform the ORF prediction and then assign function to a set of translated sequences by homology against well-curated databases of both protein and conserved domains. Finally, mine the functional and taxonomical information obtained from the search results based on the target sequences.

An alternative to avoid dealing with local software and computational resources is web portals such as Galaxy (Goecks et al., 2010), MG-RAST (Meyer et al., 2008), and IMG-M portal (Markowitz et al., 2012). These web servers are dedicated to perform taxonomical and functional analysis of metagenomes via a graphical user-friendly interface (Table 2). Unfortunately, these portals sometimes are saturated and the analysis parameters are not customizable. Finally, the internet bandwidth to transfer very large datasets could be a bottleneck for some users.

##### Metabolic pathway reconstruction

Pathway reconstruction of the metagenome data is one of the annotation goals. The concept of metabolic pathway in microbial ecology should be understood as the flow of information through different species. Therefore, the term “inter-organismic meta-routes” or “meta-pathways” has been proposed for this kind of analysis (De Filippo et al., 2012).

In order to perform a reliable metabolic reconstruction, a good functional annotation should be achieved in the first place. This has to be used to find each gene in an appropriate metabolic context, filling missing enzymes in pathways and find optimal metabolic states to perform the best pathway reconstructions. Examples of programs available are MinPath (Ye and Doak, 2009) and MetaPath (Liu and Pop, 2010). Both use information deposited in KEGG (Ogata et al., 1999) and MetaCyc (Caspi et al., 2014) repositories (Table 2).

However, most of the metabolic information comes from model organisms, but not all the enzymes or pathways are conserved among all species or environments. That is why most of the current platforms fail in metabolic reconstruction of variant pathways (de Crécy-Lagard, 2014) and most are designed to analyse single genomes.

A web service implementation by KEGG for metagenome analysis is GhostKOALA (Kanehisa Laboratories; http://www.kegg.jp/ghostkoala/). It relates taxonomic origin with their respective functional annotation, and the user is able to visualize metabolic pathways from different taxa in the same map.

Metabolic pathway reconstruction could be completed with information provided by the data context such as gene function interactions, synteny, and copy number of annotated genes to integrate the metabolic potential of consortium.

##### Bottlenecks in functional annotation: The ORFans problem

There are some relevant issues to consider in the whole metagenome shotgun sequencing annotation. Protocols based on sequence similarity searching assume that each read will be mapped to a homologous gene of some closely related species. However, depending on the database quality and size, different results could be obtained. For example, if direct DNA searches are performed, then it is probable to get matches against intergenic regions or non-coding genes (as a tRNA). In addition, alignments could retrieve best hits from a sequence in a potentially distant genome (Carr and Borenstein, 2014), affecting the taxonomic annotation if the search results are used for this endeavor (i.e., MEGAN).

In spite of the annotation method, it is known that metagenomes will have around 50% of protein sequences with no annotation or unknown function (referred as ORFans). This percentage increases when the species richness is high in the community. ORFans can be classified into three categories: (1) spurious genes produced by errors in the gene prediction; (2) genes with homology at secondary or tertiary structure level but not at nucleotide sequence level, or (3) real new genes with no homology to other genes, hence with unknown functions.

An option to deal the ORF prediction errors is to use the rate of possible non-synonymous and synonymous substitutions (ka/ks) as a criterion to select probable genes. If *ka*/*ks* value is close to 1, then it indicates that such sequence is not under selective pressure, suggesting a low probability to code for a real protein (Yooseph et al., 2008). To confirm a candidate for a novel gene, the appropriate strategy should include a *de novo* secondary and tertiary structure predictions using tools like I-TASSER (Yang et al., 2015), QUARK (Xu and Zhang, 2012), or RaptorX (Källberg et al., 2012) and perform a protein structure comparison using tools like STRAP (Gille et al., 2014). Nevertheless, this will reveal the protein tertiary structure but not necessarily its function. In fact, from more than six millions of putative enzymes identified by 454-sequencing in metagenome projects, only less than a few hundred proteins have a reliable functional annotation (Guazzaroni et al., 2010). Finally, the best way to confirm novel genes or discover new functions is through experimental procedures such as heterologous expression, biochemical characterization, and proteomics.

Pseudogenes are also a problem in metagenome functional annotation, and they could represent up to 35% in prokaryotic genomes (Liu et al., 2004). To address this annotation challenge, there are databases like BactPepDB (Rey et al., 2014) and Pseudogene.org for short sequences and pseudogenes of prokaryotic and eukaryotic organisms (Karro et al., 2007). A search in such databases before further analysis could be useful to discard non-coding sequences.

## Comparative metagenomics

In either of the metaprofiling or shotgun sequencing, the species richness or OTUs profiling could be contrasted among samples based on species diversity comparison (beta-diversity).

Two types of beta-diversity indices, such as incidence type and abundance type, could be used. The former, such as Jaccard and Sørensen indices, treats the common and rare species equally and just compares the number of shared and unique taxa between the samples. The abundance-type index contemplates abundance similarity, thereby treating individuals not species equally; some examples are the Morisita-type and Bray–Curtis dissimilarity indices (Chao et al., 2006). Such indexes are affected by sampling size. An excellent review of beta-diversity fundamentals were done by Tuomisto (2010). Alternatively, UniFrac is a method for comparing microbial communities through phylogenetic distance information contained in marker genes as the 16S ribosomal rRNA (Lozupone and Knight, 2005). This method has been well accepted in metagenomics pipelines and implemented in some R-Bioconductor packages such as phyloseq (McMurdie and Holmes, 2013) and metagenomeSeq (Paulson et al., 2013). The latter implemented a novel algorithm for normalization as alternative to rarefaction.

In metaprofiling analysis, some modular pipelines such as Mothur and QIIME are capable of analysing raw reads and performing taxonomical annotation. In addition, they can compute sample comparisons and the calculation of some indexes mentioned in the Section Concepts of Microbial Diversity and Species Richness. In order to improve diversity estimation, a lot of specialized software have been developed (Table 2) like ProViDE, which is designed for viral diversity estimation (Ghosh et al., 2011).

For whole metagenome shotgun projects, where gene protein coding information is available, functional comparative metagenomics is possible. It is based on identifying differential feature abundance (pathways, subsystems, or functional roles) between two or more conditions following a statistical procedure with some normalization step (Rodriguez-Brito et al., 2006; Pookhao et al., 2015). Some useful tools to perform robust comparative functional metagenomics are Parallel-meta and MEGAN. Other more specialized software are capable of returning graphical representations of metabolic abundances and taxonomic correlations as heatmaps or PCA plots of communities cluster genes. Two examples that compares metabolic pathways are ShotgunFunctionalizeR, which use a binomial and hypergeometric test to perform comparisons (Kristiansson et al., 2009), and MetaPath, a tool implemented in Perl that identifies and compares differentially abundant pathways in metagenomes (Liu and Pop, 2011).

## The neglected world of eukaryotes in metagenomics

Eukaryotes play important roles in almost all ecological niches in the earth; however, the study of eukaryotic domain is mostly biased toward animals, plants, and fungi, thereby resulting in a narrow view of the great eukaryotic diversity. Microscopic eukaryotes (regularly named protists) are the real bulk of most of the eukaryotic lineages (Burki, 2014). Microeukarya are poorly studied, but it is estimated that around 10% percent of protrist species are already described and were found in the ocean (Mann and Droop, 1996; Norton et al., 1996). Meanwhile, a 1.2–10 million species have been predicted as host-associated protista from which only 6000 have been reported (Burki, 2014).

Studying these organisms by NGS techniques has been a challenge because they are not well represented in the sequence databases. The lack of reference eukaryotic genomes is due in part the difficulty of their genome assembly and annotation (Gilbert and Dupont, 2011). In spite of the lack of information, it is important to remark the importance of microeukaryotes in the environment. They are responsible for CO_2_ fixing in the oceans, and they are the principal organic matter degraders in soils, and some of them are symbionts of other eukaryotes (Burki, 2014).

Diversity studies of the “eukaryotome” have been done using 18S rRNA gene amplicons (Andersen et al., 2013), and some programs include tools to analyse them such as Parallel-meta and QIIME, which have an option for mapping reads against eukaryotic Silva small ribosomal subunit (SSU) database. The SSU is commonly used for diversity analysis as universal phylogenetic marker for eukaryotic genes, but there are issues to reach a species classification level due to their little variation that limits the taxonomical position, especially for some fungi and protists (Schoch et al., 2012).

Nowadays, new strategies have been developed based on other phylogenetic markers to evaluate the eukaryotic fraction in a sample. The LSU or ITS regions are good alternatives to classify organisms at the species level with high accuracy. Ecologists interested in analysing the eukaryotic fraction are using NGS platforms like the Ion Torren PGM or the Illumina MiSeq sequencers, which generate 400 bp single reads or 300 bp paired end reads, respectively (Table 1). Both platforms deliver enough yield to perform the analysis of LSU or ITS amplicons at a very high depth (Lindahl and Kuske, 2013; Hugerth et al., 2014; Tonge et al., 2014).

Regarding the metabolic association of eukaryotic genes in a certain pathway, it can be a greater challenge than bacterial annotation. Eukaryotic genomes are typically 6–10 times larger than the average bacterial genome (about 3–5 Mb) size, plus they can have different genome ploidy states. It is worthy to mention that the eukaryotic genes contain introns, which may have differential splicing patterns under particular environmental conditions, thereby increasing the amount of products (isoforms) with different functions to annotate. Moreover, high percentage of intergenic non-coding sequences that are represented differently in a shotgun sequenced metagenome can represent a problem if they were not assembled correctly leaving them out of their gene context. A strategy to further characterize coding regions in a eukaryotic metagenome is to isolate some mRNA to perform a metatranscriptomics analysis. Enriched mRNA from eukaryotic organisms (Qi et al., 2011; Keeling et al., 2014) can be *de novo* assembled or mapped to related reference genomes in order to elucidate the functions from these transcripts.

## Concluding remarks

Here, we have reviewed the evolution of Microbiology into Metagenomics to describe exhaustively a microbial community in terms of taxonomic diversity and metabolic potential. Metagenomics allows us to discover new genes and proteins or even the complete genomes of non-cultivable organisms in less time and with better accuracy than classical microbiology or molecular methods. However, there are no standard methods or universal tools that can answer all of our questions in metagenomics. In fact, the lack of standards reduces the reproducibility and comparison between similar projects, making metagenomics a case by case study. It is noteworthy that each metagenome project has specific requirements depending on its experimental design, and hence, the sequencing technology and computational tools should be chosen carefully. In spite of the serendipity that is present in science, we have to bear in mind that the experimental design is the most important part and should fit each project objectives in order to reach them and answer the biological question behind the project.

A metagenome usually represents a snapshot of a community at a certain time when its DNA is obtained. As mentioned, a good experimental design is necessary to explore the complete population dynamics by combining different approaches like culture methods, DNA and RNA analysis, protein studies, and if possible, the metabolic profile. Consequently, integration of several tools to microbiology (such as molecular biology, genetics, bioinformatics, and statistics) is necessary to answer the questions related to microbial diversity and ecology in a greater extent.

In our opinion, the development of more bioinformatics tools for metagenomics analysis is necessary, but the experience of scientists to manipulate such tools and interpret their results is the key to a sensible biological conclusion. The bioinformatics expertise is a necessity, as the sequencing platforms are delivering a massive yield at a very low cost, increasing the amount of information to analyse. Finally, the near future challenge will reside in the manipulation and analysis of the data deluge and how we can interpret them in a more integrative way that could reflect the biodiversity present in our world.

### Conflict of interest statement

The authors declare that the research was conducted in the absence of any commercial or financial relationships that could be construed as a potential conflict of interest.
